# Analysis of inter-system variability of systolic and diastolic intraventricular pressure gradients derived from color Doppler M-mode echocardiography

**DOI:** 10.1038/s41598-020-64059-4

**Published:** 2020-04-28

**Authors:** Amir Hodzic, Odile Bonnefous, Hélène Langet, Walid Hamiche, Laure Chaufourier, Francois Tournoux, Paul Milliez, Hervé Normand, Eric Saloux

**Affiliations:** 1 Normandie Univ, Unicaen, CHU Caen Normandie, Department of Clinical Physiology, Inserm Comete, 14000 Caen, France; 2 Normandy univ, Unicaen, CHU Caen Normandy, Cardiology department, Caen, France; 3 Philips Research, Medical Imaging (Medisys), Suresnes, France; 4 Research Center of the Hospital of the University of Montreal (Centre de Recherche du Centre Hospitalier de l’Université de Montréal), Montreal, Canada

**Keywords:** Physiology, Cardiology, Applied physics

## Abstract

Assessment of intraventricular pressure gradients (IVPG) using color Doppler M-mode echocardiography has gained increasing interest in the evaluation of cardiac function. However, standardized analysis tools for IVPG quantification are missing. We aimed to evaluate the feasibility, the test-retest observer reproducibility, and the inter-system variability of a semi-automated IVPG quantification algorithm. The study included forty healthy volunteers (50% were men). All volunteers were examined using two ultrasound systems, the Philips Epiq 7 and the General Electric Vivid 6. Left ventricular diastolic (DIVPG) and systolic (SIVPG) intraventricular pressure gradients were measured from the spatiotemporal distribution of intraventricular propagation flow velocities using color Doppler M-mode in standard apical views. There was good feasibility for both systolic and diastolic IVPG measurements (82.5% and 85%, respectively). Intra and inter-observer test-retest variability measured with the intraclass correlation coefficient were 0.98 and 0.93 for DIVPG respectively, and 0.95 and 0.89 for SIVPG respectively. The inter-system concordance was weak to moderate with Lin’s concordance correlation coefficient of 0.59 for DIVPG and 0.25 for SIVPG. In conclusion, it is feasible and reproducible to assess systolic and diastolic IVPG using color Doppler M-mode in healthy volunteers. However, the inter-system variability in IVPG analysis needs to be taken into account, especially when using displayed data.

## Introduction

Left ventricular (LV) systolic contraction and early diastolic active relaxation generate forces that transmit regional pressure differences to the LV cavity. Hemodynamic investigations have demonstrated that the assessment of intraventricular pressure gradients (IVPGs) provides determinant insights in cardiac function^1–4^. LV ejection is facilitated by a positive systolic intraventricular pressure gradient (SIVPG) between the apex and the outflow tract. The SIVPG increases during physical exercise and inotropic stimulation, whereas it is attenuated by infusion of betablockers^1,2^. During the early diastole, LV active relaxation generates suction by developing a local negative diastolic pressure gradient between the mitral valve and the LV apex (DIVPG). This mitral to apical driving pressure was closely related to the rate of LV relaxation^5^, and it was shown to be sensitive to inotropic stimuli, loading variations, and acute myocardial ischemia^5,6^. For the past two decades, experimental and human studies have demonstrated the validity of color Doppler M-mode echocardiography to estimate non-invasively the instantaneous local pressure differences into LV during ejection and diastolic filling^7–13^. These authors have confirmed the close relationship between color Doppler-derived systolic and diastolic IVPGs and invasively measured indices of LV contractility and relaxation^14–16^. Several studies have evaluated the clinical applications of IVPGs assessment using color Doppler M-Mode in healthy subjects and patients with cardiomyopathies. Heart failure with preserved and reduced ejection fraction was associated with a loss of adrenergic augmentation of DIVPGs under pharmacological and physical stress^17–19^. These observations suggested that heart failure is associated with a reduced ability to generate an effective ventricular suction in such stress conditions, essential to ensure rapid LV filling without increase in atrial pressure. Similar observations of reduced LV apical suction in heart failure patients were reported in resting-state^20^. Inversely, reduction of left ventricular obstruction after ethanol septal reduction in a small group of patients with hypertrophic cardiomyopathy was associated with increased DIVPGs, suggesting an improvement in diastolic suction force^12^. The same authors have also demonstrated that the inability to augment DIVPGs in heart failure was strongly correlated with peak VO_2_^18^.

These echocardiographic studies have used the same approach based on the Bernoulli equation to estimate non-invasively the instantaneous local pressure gradient from the spatiotemporal distribution of blood velocities along the LV long axis. However, these studies have used different ultrasound scanners for data acquisitions and different processing chains of the blood flow velocity data. To date, only one study addressed the impact of color-Doppler scanner resolutions when calculating IVPGs from color Doppler M-mode^21^. This point is particularly relevant for clinical quantitative processing of color Doppler-derived IVPGs to characterize cardiac function in patient cohorts. The temporal, spatial and velocity resolutions differ between ultrasound vendors, some of whom restrict the access of raw proprietary velocity data. The lack of studies evaluating the inter-system reliability of a standardized method for IVPG quantification might limit the clinical interest of physicians. The present study aimed to assess the feasibility, the test-retest observer reproducibility, and the inter ultrasound system variability of systolic and diastolic IVPG measurements, using a semi-automated algorithm based on velocities extracted from color Doppler M-mode image file.

## Methods

### Study design

Forty healthy volunteers (50% were men), aged between 18 and 35 years old, were prospectively enrolled among medical students at the university hospital center of Caen (France). The upper age limit was set at 35 years-old to limit the prevalence of cardiovascular diseases that could impact myocardial function. Participants were not taking any medication, and cardiovascular disease was ruled out by history, clinical examination, electrocardiogram, and echocardiography. Subjects with metabolic and chronic extracardiac diseases were excluded. All volunteers referred for a standard echocardiographic examination were recruited on the condition of an acceptable apical ultrasonic view. The study protocol was approved by the institutional ethical committee (Comité de Protection des Personnes Nord Ouest III – ID: A16-D11-VOL.27), and written informed consent was obtained from all participants. The medical procedures of this trial are in line with the most recent recommendations of the Helsinki Declaration.

To assess the inter-system agreement of IVPG measurements, each participant was sequentially examined by the same sonographer on two different ultrasound systems: the Philips Epiq 7 equipped with an X5-1 transducer, and the General Electric (GE) Vivid 6 equipped with an M4S transducer. The ultrasound systems were identified randomly as system A and system B in this publication. Ultrasound machines were arranged on the same side of the examination bed. The sonographer position, scanning right-handed, did not change between examinations. For each volunteer, color Doppler M-mode acquisitions for systolic and diastolic IVPG assessment were performed consecutively in the same session using both ultrasound systems (time required to switch between probes from system A to system B). All examinations were made in the left lateral decubitus position. Left ventricular inflow and outflow recordings using color Doppler M-mode were obtained from all subjects on three consecutive cardiac cycles in apical four and five-chamber views during a brief apnea. Arterial blood pressure was measured during the echocardiographic study. Heart rate was monitored during the examinations. Two sonographers were able to perform echocardiographic examinations. The first sonographer (AH) was well trained in IVPG acquisitions. The second sonographer (LC) was less experienced. All participants were provided the option to choose the gender of the sonographer who would perform their examinations. Intra and inter-observer test-retest variability for IVPG measurements were determined on ten randomly selected subjects scanned by the two sonographers, who acquired each two independent SIVPG and DIVPG data sets, using the same machine. The sonographers analyzed their own data sets in a blinded fashion. For each data set, peaks IVPG (systolic and diastolic) were obtained as the average value of three consecutive cardiac cycles.

All color Doppler M-mode acquisitions were stored as Dicom files, which contain raw and processed data displayed in Joint Photographic Experts Group (JPEG) format. IVPGs were measured using our independent software algorithm coded in Matlab (version R2017b) from displayed data.

### IVPG assessment by color Doppler M-Mode

Color Doppler M-mode provided both a spatiotemporal map of greyscale anatomical information and a spatiotemporal map of color-encoded blood velocity distribution $v=v(s,t)$ in the direction of the ultrasound streamline, where $v$ represents the velocity, $s$ represents the spatial dimension defined along the streamline, and $t$ represents the time. For an inviscid flow without body forces, the equations of the fluid mechanics relate the change of velocity along a streamline (convective acceleration) and the change of velocity with time (inertial acceleration) to the change in pressure along the same streamline:

$$\frac{\partial p}{\partial s}=-\,\rho \bullet \left(v\bullet \frac{\partial v}{\partial s}+\frac{\partial v}{\partial t}\right)$$

**Figure 1:**
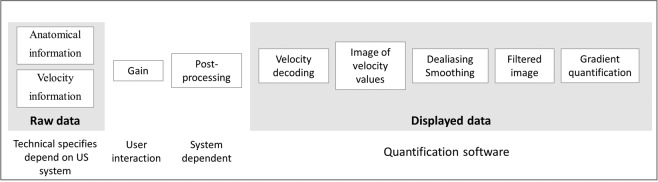
Image acquisition and processing diagram.

**Figure 2:**
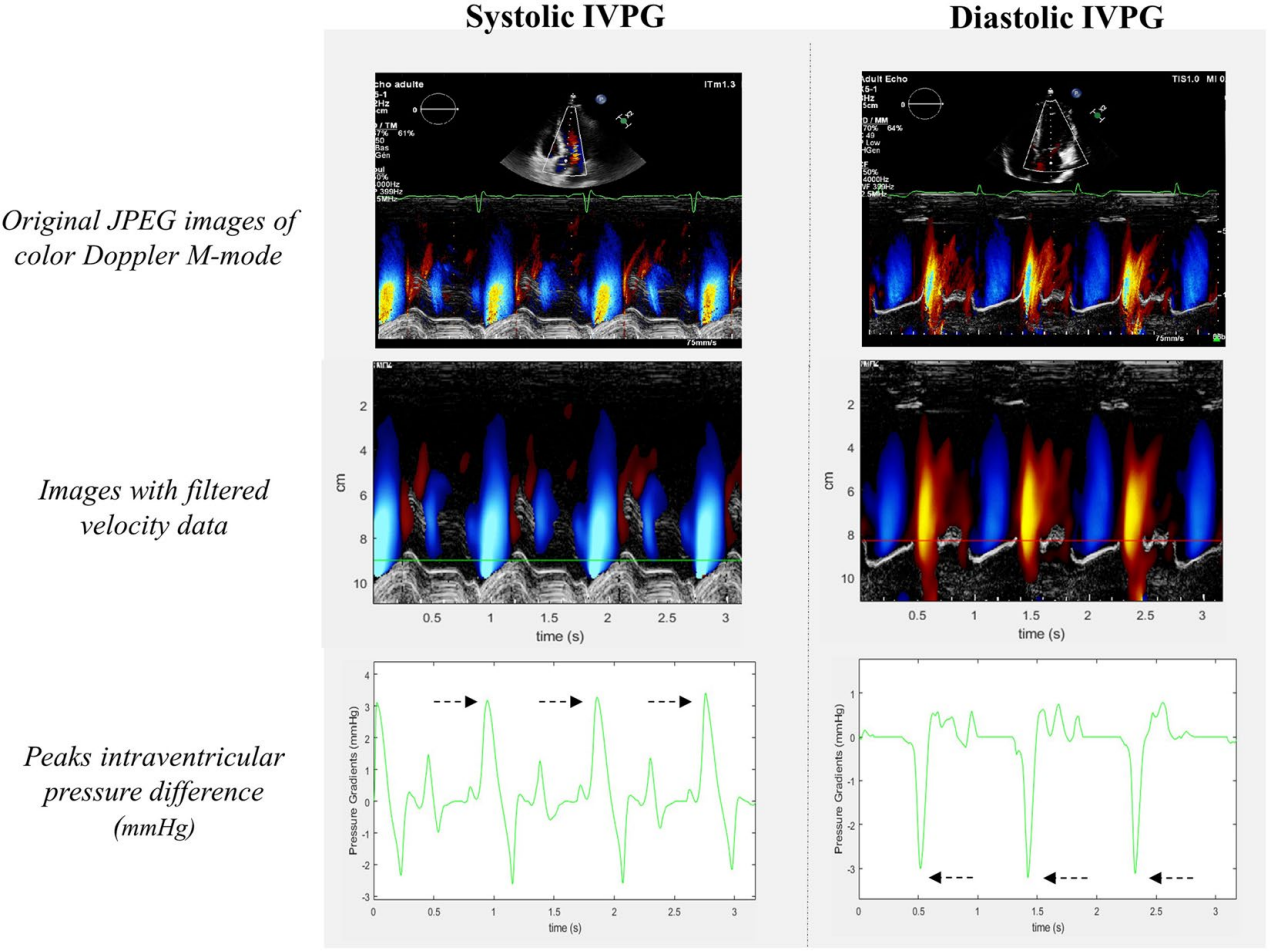
Color Doppler M-mode recordings and processing of systolic and diastolic intraventricular pressure gradients (IVPG). The spatiotemporal velocity profile along the streamline from the left ventricular apex to the aortic annulus in systole and from the mitral annulus to apex in diastole, acquired by color Doppler M-mode, was used to compute systolic and diastolic IVPG after an automated dealiasing and smoothing process. Anatomic streamline markers were positioned at the mitral (red line) and aortic (green line) annulus. Intraventricular pressure difference curves were obtained during the entire cardiac cycle, using the Bernoulli equation. Peaks systolic and diastolic IVPG (black arrows) were averaged on three consecutive cycles.

The velocity data were extracted from the displayed image by using the color encoding given by the Look-Up Table (LUT). To enable further quantification, the velocity data were processed with dealiasing and smoothing (Fig. 1). We used a simple Gaussian kernel to estimate the variance of the color and to smooth the velocity for adequate temporal and spatial derivative estimation. The typical kernel is defined by temporal sigma = 5 * delta t and spatial sigma = 2 * delta z, where delta t and delta z are the time and space sampling intervals. As a matter of fact, the final result quality depends on the resolution of the data. The temporal derivative of the velocity was simply computed after smoothing and the two terms of the Euler’s equation were integrated along the M-mode to calculate diastolic and systolic IVPG (Fig. 2), as previously validated^8,10,11^. The position of the mitral and aortic annulus had to be defined to design the integration limits. It was automatically positioned from the echo M-mode and could be manually corrected by the reader if the result was not judged as correct. Examinations that did not meet the quality criteria were not processed. The internal validation of image quality before processing was: 1/optimal alignment of the scan line with the direction of blood flow as described by Greenberg *et al*.^8^; 2/complete color Doppler flow map through the streamline; 3/presence of anatomical landmarks of LV in the color Doppler sector; 4/absence of flow obstruction.

The temporal resolution was set by default to acquire three successive complete cardiac cycles to guarantee the stability of the averaged IVPG measure. Consequently, the displayed image resolutions for system A were 285 Hz for the temporal resolution and 0.24 mm for the spatial resolution, and for system B 180 Hz for the temporal resolution and 0.42 mm for the spatial resolution. To study the potential impact of temporal sampling on the measure of pressure gradient differences, we assessed SIVPG and DIVPG at different temporal displayed resolution settings (95 Hz, 190 Hz, 285 Hz, 380 Hz, and 570 Hz) on ten randomly selected subjects scanned with system A. For each subject, peaks IVPG were averaged during the same period of three cardiac cycles in systole and diastole.

**Figure 3:**
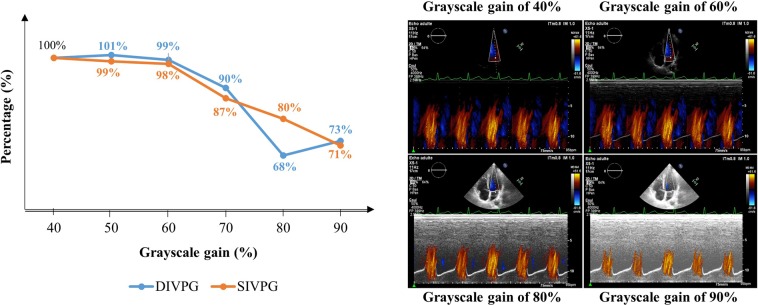
Adjusting the grayscale gain at 60% provided the best compromise between color flow mapping and 2D gray-scale definition level of the left ventricular apex and base. The graphic (left panel) shows that a grayscale gain adjustment above 60% was responsible for a growing decrease in systolic (red line) and diastolic (blue line) intraventricular pressure gradients (IVPG). Data are expressed as a mean percentage decrease of peak IVPG from the initial value measured at 40% of the grayscale gain and averaged on fifteen different color Doppler M-mode acquisitions in systole and diastole. The right panel shows diastolic IVPG acquisitions made at a different grayscale gain (40%, 60%, 80%, and 90%).

The greyscale gain was adjusted to optimize the color flow mapping on the displayed image. For that, we tested a range of the grayscale gain on 15 color Doppler M-mode acquisitions for both systolic and diastolic gradients. For each acquisition, the gain was increased by steps of 10% from 40% up to 90%. Figure 3 (left panel) shows that for a grayscale gain above 60%, we observed a loss of color Doppler flow velocity information responsible for a decrease in peaks IVPG compared to the initial value obtained with the lowest grayscale gain (expressed as a percent). Acquisitions made at a grayscale gain of 60% provided the best compromise between color flow mapping and 2D gray-scale definition level of LV anatomy (Fig. 3, right panel). In the present study, the grayscale gain was set at 60% for all color-Doppler M-mode acquisitions.

### Statistical analysis

Statistical analysis was conducted using Medcalc version 13 (MedCalc Software, Ostend, Belgium). All variables were normally distributed (Shapiro-Wilk test) and expressed as mean ± SD. IVPG measurements were expressed as absolute values. The agreement between the ultrasound system acquisitions was estimated by calculating the mean and SD of the difference between system A and system B (Bland and Altman^22^), as well as Lin’s concordance correlation coefficient (CCC)^23^. Intra and inter-observer test-retest variability for SIVPG and DIVPG measurements were determined using the intraclass correlation coefficient (for average fixed observers) with a 95% confidence interval, and the coefficient of variation ± SD. A paired t-test was used to analyze the significance of the biases in IVPG measurements between systems. Mean values of IVPGs between men and women were compared using unpaired t-test. The level of significance was defined as P < 0.05.

## Results

The mean age of the enrolled volunteers was 25.5 ± 3.7 years old (25.3 ± 3.5 years for men *vs*. 25.7 ± 4.1 years for women, P = 0.65). The overall body mass index was 22.2 ± 2.3 kg/m². The mean arterial blood pressure was 86 ± 8.8 mmHg. The heart rate remained stable during system A and system B examinations (74 ± 13.3 beats/min *vs*. 76 ± 12.8 beats/min, P = 0.42). No gender differences were observed in arterial blood pressure nor heart rate. All subjects were in sinus rhythm. Cardiac function and structure were normal, without any valvular diseases.

### Feasibility and reproducibility of IVPG measurements

Among the 40 volunteers included in the study, 7 examinations for SIVPG acquisitions and 6 examinations for DIVPG acquisitions did not meet the internal validation of image quality on both systems and were excluded. Thus, the overall feasibility of IVPG acquisitions was 82.5% for SIVPG acquisitions (33/40 subjects) and 85% for diastolic acquisitions (34/40 subjects). The feasibility was different according to the experience of the sonographer who performed the examination. For the well-trained operator in IVPG acquisitions (AH), who scanned 22 subjects during the study, the feasibility was up to 91% (20/22 subjects) for both systolic and diastolic color Doppler acquisitions. Whereas, for the less-trained operator (LC), who scanned 18 subjects during the study, only 72% (13/18 subjects) of SIVPG and 78% (14/18 subjects) of DIVPG acquisitions met the validation criteria. Examples of IVPG acquisitions that did not meet the validation criteria are illustrated in Supplementary Fig. 1.

Regarding intra-observer test-retest variability, for DIVPG the intraclass correlation coefficient was 0.98 [IC 95%: 0.94–1], and the coefficient of variation was 5.2 ± 0.1%. For SIVPG the intraclass correlation coefficient was 0.95 [IC 95%: 0.83–0.99], and the coefficient of variation was 6.3 ± 0.2%. Regarding inter-observer test-retest variability, for DIVPG the intraclass correlation coefficient was 0.93 [IC 95%: 0.83–0.97], and the coefficient of variation was 10.5 ± 0.3%. For SIVPG the intraclass correlation coefficient was 0.89 [IC 95%: 0.74–0.95], and the coefficient of variation was 9.3 ± 0.3%.

### Analysis of inter-system variability

**Figure 4:**
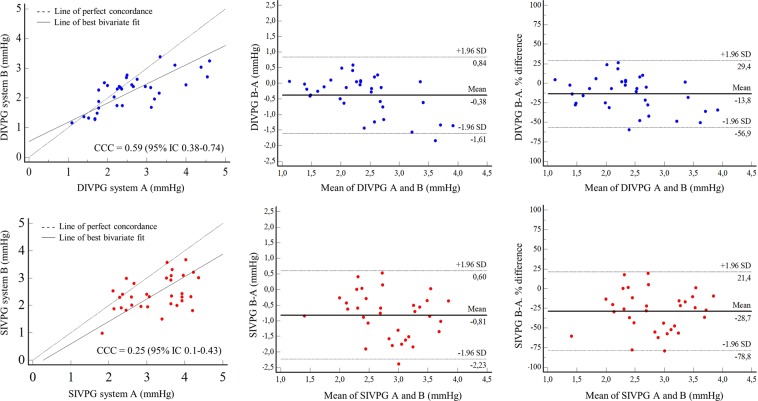
Lin’s concordance correlation coefficient (CCC) and Bland Altman analysis for diastolic (DIVPG) and systolic (SIVPG) intraventricular pressure gradients measurements between system A and system B. The line of perfect concordance is the line of identity (*y = x*); the reduced major axis corresponds to the regression line between the two measurements.

Table 1 shows the mean values of peaks SIVPG and DIVPG obtained with the two ultrasound systems A and B in the overall population. Both DIVPG and SIVPG measurements were significantly different between system A and system B. Lin’s CCC for DIVPG was 0.59 (IC 95% 0.38–0.74) between system A and system B (Fig. 4). For SIVPG, the concordance was weaker between system A and system B with Lin’s CCC = 0.25 (IC 95% 0.1–0.43) (Fig. 4). As shown by Bland-Altman plots, a mean bias of −13.8% was observed for DIVPG estimates and −28.7% for SIVPG estimates between system A and system B (Fig. 4). No gender difference was observed for overall DIVPG measurements (2.51 ± 0.75 mmHg for men *vs*. 2.24 ± 0.83 mmHg for women, P = 0.17). Overall SIVPG measurements were significantly higher among men compared to women (3.11 ± 0.75 mmHg *vs*. 2.55 ± 0.71 mmHg respectively, P = 0.005).

**Table 1:** Mean values of systolic (SIVPG) and diastolic (DIVPG) intraventricular pressure gradients measured in the overall population with the two ultrasound systems A and B.

	System A	System B	P-value
**SIVPG** (mmHg)	3.24 ± 0.74	2.43 ± 0.61	P > 0.0001
**DIVPG** (mmHg)	2.58 ± 0.91	2.2 ± 0.59	P = 0.001

**Figure 5:**
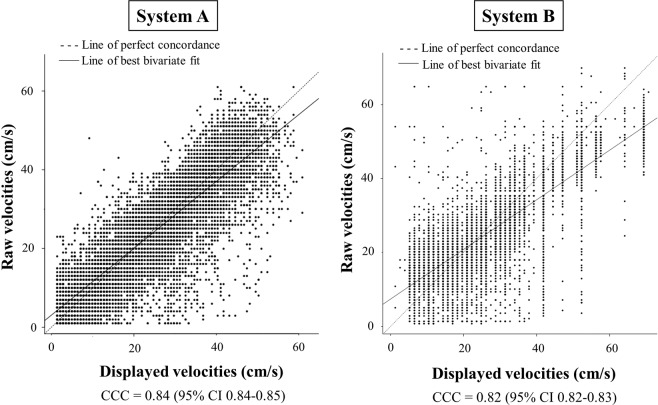
Lin’s concordance correlation coefficient (CCC) between the pixel-to-pixel velocity value comparison extracted from the raw proprietary velocity data and the displayed velocity data estimated from the look-up table, on one sample of color-Doppler M-mode acquisition for both systems A and B. The line of perfect concordance is the line of identity (y = x); the reduced major axis corresponds to the regression line between the two measurements.

To analyze if the displayed blood flow velocities were not more degraded by one of the two systems, we were able to test the concordance between the raw proprietary velocity data and the displayed velocity data estimated from the LUT on both systems for one set of color Doppler M-mode diastolic acquisition. The results are reported in Fig. 5. Both systems showed similar concordance between displayed and raw velocity data. The Lin’s CCC was 0.84 (95% CI 0.84–0.85) for system A and 0.82 (95% CI 0.82–0.83) for system B. Displayed velocity data are undersampled compared to raw velocity data in both systems. The raw data are coded on 8 bits and exhibit 256 values, whereas the decoded LUT allowed allocating only 150 shades for system A and 110 shades for system B.

### The effect of temporal displayed resolution on inter-system variability

**Figure 6:**
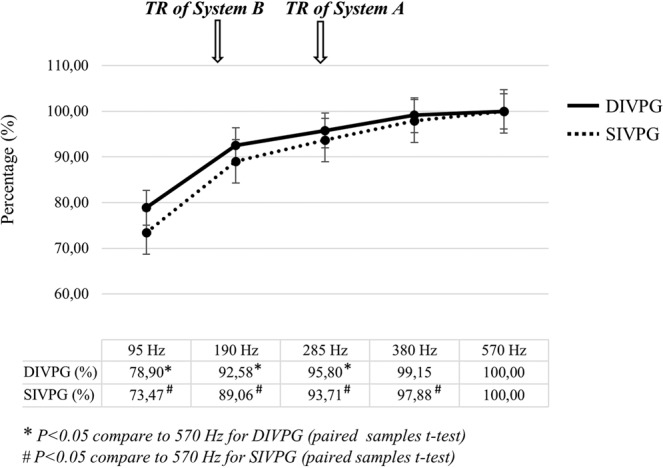
Effects of temporal displayed resolution changes on systolic (SIVPG) and diastolic (DIVPG) intraventricular pressure gradients measurements. Peaks SIVPG and DIVPG were assessed at different temporal displayed resolution settings (95 Hz, 190 Hz, 285 Hz, 380 Hz, and 570 Hz) in ten randomly selected subjects scanned with system A. Data are expressed as a mean percentage decrease of peaks SIVPG and DIVPG from the initial value measured at 570 Hz. Arrows indicate the default temporal displayed resolutions of system A and system B.

The results are shown in Fig. 6. As expected, peaks SIVPG and DIVPG decreased with the reduction of temporal displayed resolution. For DIVPG, a significant diminution was observed when the temporal displayed resolution was set lower than 380 Hz. However, up to 285 Hz, the mean reduction of DIVPG was small (−2.7% ± 2.4% compared to the measure at 570 Hz). A greater underestimation was observed for a temporal displayed resolution below than 285 Hz (mean difference of −5.8% ± 3.3% at 190 Hz and −26.5% ± 10.5% at 95 Hz). For SIVPG, a significant but small underestimation was observed with a temporal displayed resolution of 380 Hz (mean difference of −2.74% ± 2.6% compared to the measure at 570 Hz). Acquisitions made at a resolution of 285 Hz and less were responsible for a growing underestimation of peaks SIVPG (mean difference of −6.5% ± 6% at 285 Hz, −10.9% ± 9.8% at 190 Hz, and −25.9% ± 12.4% at 95 Hz).

## Discussion

This study confirmed that IVPG quantification using color-Doppler M-mode echocardiography, previously validated in experimental and human studies^8,9,11,13^, is feasible and highly reproducible. However, we observed an inter-system variability, which might be mostly explained by differences in the temporal displayed resolutions of processed velocity data between the two ultrasound systems.

In the present study, we used a semi-automated software for processing IVPGs that resulted in high intra- and inter-observer test-retest reproducibility. Although IVPG assessment requires an experienced operator to correctly acquire color Doppler M-mode velocity profiles during systole and diastole, as suggested by a higher rate of feasibility for the well-trained operator in IVPG acquisitions, the overall feasibility for both systolic and diastolic IVPG measurements was acceptable. The choice of the operator did not significantly influence the concordance between systems A and B measurements for both SIVPG and DIVPG (data not shown). If the color Doppler M-mode acquisitions fulfilled the required quality criteria for IVPG assessment, the post-processing did not appear to be influenced by the operator’s experience, which was confirmed by our test-retest analysis. These results confirmed the good inter-reader reproducibility of echocardiographic diastolic and systolic IVPG measurements previously reported^12,16,18^. Ohara *et al*.^19^ have shown the clinical utility and feasibility of DIVPG assessment in a large cohort of 166 patients with diastolic dysfunction undergoing stress echocardiography. Yet, the authors did not report whether there have been patients excluded from the study due to insufficient image quality to assess color-Doppler derived DIVPGs. Based on these observations, and in contrast with the alias-based slope method for measuring the color M-mode flow propagation velocity (Vp) which is no more recommended in the routine assessment of LV diastolic function due to low feasibility and reproducibility^24^, DIVPGs seem to provide a clinically applicable and reliable evaluation of LV suction.

The present study analyzed for the first time the inter-system variability of color Doppler M-mode derived IVPG assessment using two of the most common echocardiographic systems in echo labs (Philips and GE). The concordance between IVPG measurements acquired with the two ultrasound systems was weak to moderate. Sources of inter-system variability for color Doppler-derived IVPGs analysis can be multiple from the color Doppler velocity data acquisition to the image post-processing. The risk of errors caused by color-Doppler scanner resolutions has been investigated^21^. Rojo-Alvarez JL. *et al*.^21^ have demonstrated the importance of the temporal resolution in the calculation of IVPGs derived from flow color Doppler. The authors have estimated by an analytical approach that IVPG calculation requires an ideal image temporal resolution of at least 3000 Hz to ensure a risk of error of less than 10% considered clinically acceptable. However, this is significantly higher than the scanner temporal resolution used (200 Hz) in the first studies that have validated the technique by comparison to direct measurements with micromanometers^8–10^. In accordance, our results showed the impact of the temporal displayed resolution on color Doppler-derived IVPG quantification.

Because raw proprietary velocity data of color flow mapping are not routinely provided by the ultrasound systems that we tested, IVPG quantification was made on the same displayed format image. In the absence of recommendations for systolic and diastolic IVPG quantifications based on displayed JPEG data obtained from color Doppler velocity profiles, we showed that to prevent a significant underestimation of IVPGs (Figs. 3 and 6) the acquisitions should be made with a grayscale gain of 60% and a temporal displayed resolution of at least 285 Hz. As described in our results, color-Doppler JPEG images obtained from system A and system B exhibited different displayed spatial and temporal resolutions that could not be homogenized. Doppler acquisitions made in clinics usually focus on getting a sufficient number of cardiac cycles to average the measurements but at the cost of reducing temporal image resolution and risking to underestimate IVPGs. More, it is not easy for clinicians to link the temporal resolution with the horizontal sweep velocity of the displayed image. The Bernoulli equation defines that the instantaneous pressure between any two points along the flow streamline ($\partial P/\partial s)$ is the net result of the inertial component ($\partial v/\partial t)$ and the convective component $(\partial v/\partial s)$. Thus, we can suppose that a temporal subsampling could be responsible for a theoretical risk of errors in the connective component estimation by missing the peaks of the maximal local flow velocities variations during the diastole and systole.

Another source of inter-system variability could also be related to the fact that IVPGs were calculated from the displayed blood flow velocity data extracted from the LUT. We ignored whether the velocity data encoded in JPEG were consistent with the raw velocity data. To test the accuracy of using displayed velocity data given by the LUT to calculate IVPGs, we analyzed the concordance between the raw proprietary velocity data and the displayed velocity data on both ultrasound systems. Our findings showed similar concordance between raw and displayed velocity data for both systems, suggesting that the use of displayed velocity data might not be responsible for major inaccuracies in IVPG measurements. However, IVPG quantification based on processed data is challenged by multivendor differences in the displayed resolution of the image.

### Clinical implications

The present study has confirmed good overall feasibility and observer reproducibility of systolic and diastolic IVPGs assessed by an automated quantification tool using displayed data in a healthy population. However, even using a standardized method for IVPG calculation we observed low inter-system concordance, which could be related to inter-system specificities in color-Doppler signal acquisition and image resolutions. These observations raise the question of more uniformity in the color Doppler acquisition process between ultrasound vendors, and larger accessibility to raw proprietary velocity data. Our results are evocative of some concerns recently highlighted about speckle tracking-derived strain. This semi-recent technique has been widely demonstrated as a sensitive marker of LV dysfunction and patient outcomes. However recent reports have pointed out its high inter-vendor variability^25^ encouraging the learned society of echocardiography to focus on homogenizing the type of stored data used to quantify left ventricular strain. We believe that a similar reflection should be carried out on measurements based on flow color Doppler data.

### Limitations

The first limitation of our study is related to the technique used for the non-invasive assessment of IVPGs. The validity of systolic and diastolic IVPG assessment based on the Bernoulli equation requires assuming a laminar intracardiac flow where the considered streamline coincides with the scanline. This assumption of a laminar flow has been previously validated in experimental and numerical studies^8,11^. Another limation is related to our population. The small number of subjects included in our study may limit the statistical analysis. Further, our results of IVPG reliability cannot be generalized to patients with systolic dysfunction and pathological ventricular remodeling which could be associated with a modulation of the spatiotemporal velocity distribution of intraventricular flows^26^. However, previous studies have shown good feasibility of IVPG assessment using color Doppler M-mode in patients with heart failure and reduced ejection fraction^13,17,18,20^. In addition, we cannot exclude that the manual correction of the mitral and aortic annulus level left to the observer’s appreciation could participate in the inter-system variability. Although we have been careful to accurately assess LV landmarks during post-treatment, a misalignment near the valvular annulus plan where the velocities are the highest could induce variability in IVPG analysis. However, our quantification tool showed excellent test-retest reproducibility. To end, among other parameters that could have been a source of inter-system variability, we were not able to evaluate the equivalence of the value of priority given to the color flow between systems. We used the threshold value that was optimized by the ultrasound vendors on their commercially available machines.

## Conclusion

Non-invasive estimation of the instantaneous intraventricular pressure differences, closely related to the LV systolic contractile forces and early diastolic relaxation, has demonstrated its clinical potential. However, IVPG assessment using color-Doppler M-mode remains confined to research. Our automated quantification tool, which could be implemented on ultrasound machines, was able to assess systolic and diastolic IVPGs with good feasibility and high observer test-retest reproducibility in healthy volunteers. The inter-system variability, related to inter-vendor differences in image resolutions, needs to be brought to the attention of clinicians who are interested in quantitative assessment of IVPGs on displayed velocity data.

## Supplementary information

Supplementary Figure 1.

## Data Availability

The datasets generated during and/or analyzed during the current study are available from the corresponding author on reasonable request.
