# Supplementary material for: Analysis of inter-system variability of systolic and diastolic intraventricular pressure gradients derived from color Doppler M-mode echocardiography
Source: Sci Rep. 2020 Apr 28;10:7180. doi: 10.1038/s41598-020-64059-4 (PMC7188811; doi:10.1038/s41598-020-64059-4)

SUPPLEMENTARY MATERIAL

Analysis of inter-system variability of systolic and diastolic intraventricular pressure gradients derived from color Doppler M-mode echocardiography.

Amir Hodzic, Odile Bonnefous, Hélène Langet, Walid Hamiche, Laure Chaufourier, Francois Tournoux, Paul Milliez, Hervé Normand, Eric Saloux.

SUPPLEMENTARY FIGURE:

Supplementary Figure 1: Examples of intraventricular pressure gradient (IVPG) acquisitions that did not meet the internal validation criteria and that were excluded from the final analysis.

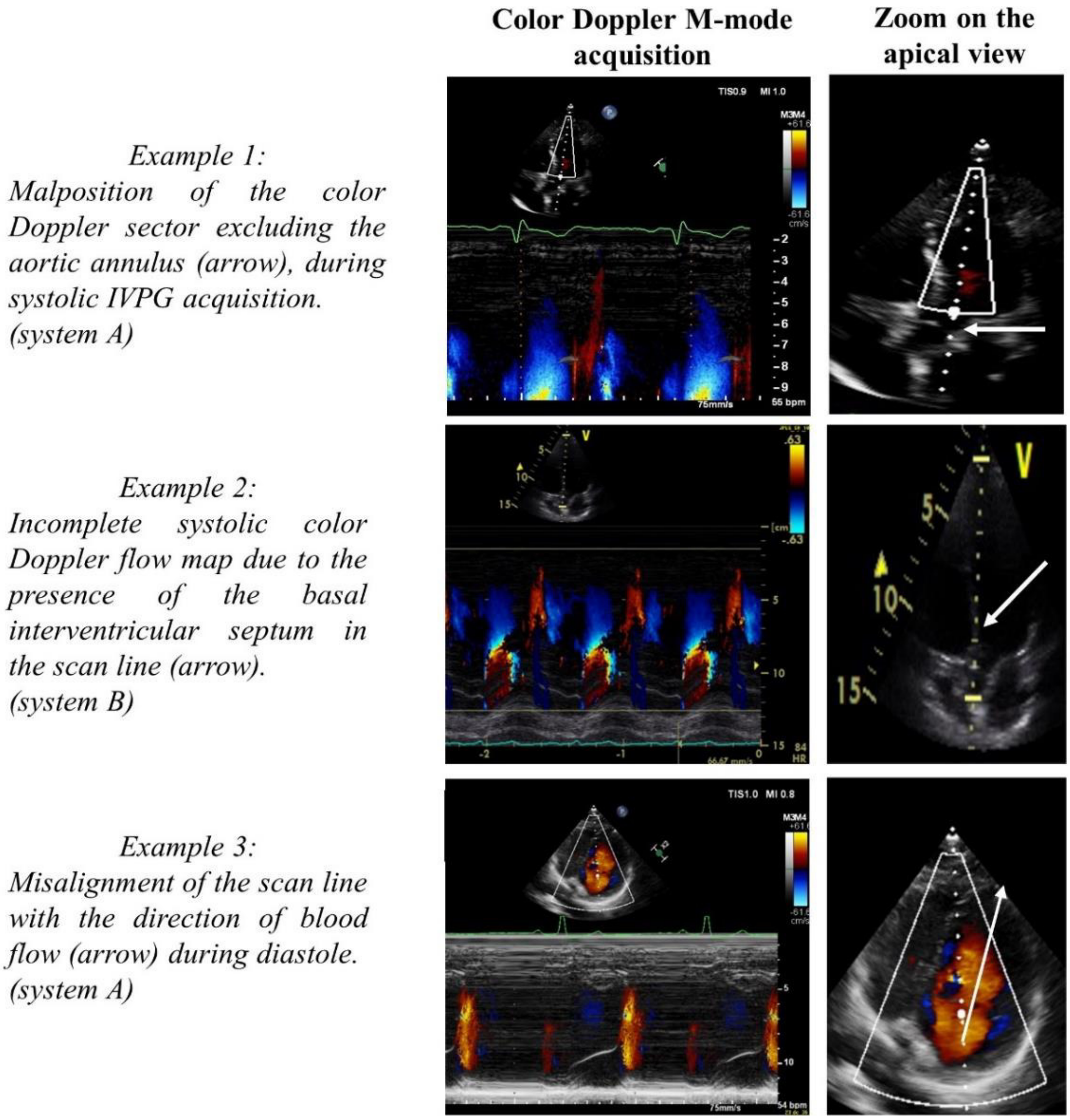

Supplement: Supplementary file 1 — Supplementary Figure 1. [file 41598_2020_64059_MOESM1_ESM.pdf]
